# What does it mean to be wild? Assessing human influence on the environments of nonhuman primate specimens in museum collections

**DOI:** 10.1002/ece3.8006

**Published:** 2021-08-09

**Authors:** Andrea R. Eller, Stephanie L. Canington, Sana T. Saiyed, Rita M. Austin, Courtney A. Hofman, Sabrina B. Sholts

**Affiliations:** ^1^ Department of Anthropology National Museum of Natural History Smithsonian Institution Washington DC USA; ^2^ Center for Functional Anatomy and Evolution Johns Hopkins University School of Medicine Baltimore MD USA; ^3^ Department of Anthropology University of Notre Dame Notre Dame IN USA; ^4^ Natural History Museum University of Oslo Oslo Norway; ^5^ Department of Anthropology University of Oklahoma Norman OK USA; ^6^ Laboratories of Molecular Anthropology and Microbiome Research University of Oklahoma Norman OK USA

**Keywords:** anthrome, captive, evolution, natural history, primates

## Abstract

**Objectives:**

Natural history collections are often thought to represent environments in a pristine natural state—free from human intervention—the so‐called “wild.” In this study, we aim to assess the level of human influence represented by natural history collections of wild‐collected primates over 120 years at the Smithsonian Institution's National Museum of Natural History (NMNH).

**Materials and Methods:**

Our sample consisted of 875 catarrhine primate specimens in NMNH collections, representing 13 genera collected in 39 countries from 1882 to 2004. Using archival and accession information we determined the approximate locations from which specimens were collected. We then plotted location coordinates onto publicly available anthrome maps created by Ellis et al. (*Global Ecology and Biogeography*, 2010, 19, 589), which delineate terrestrial biomes of human population density and land use worldwide since the 1700s.

**Results:**

We found that among primates collected from their native ranges, 92% were from an environment that had some level of human impact, suggesting that the majority of presumed wild‐collected primate specimens lived in an environment influenced by humans during their lifetimes.

**Discussion:**

The degree to which human‐modified environments may have impacted the lives of primates currently held in museum collections has been historically ignored, implicating unforeseen consequences for collection‐based research. While unique effects related to commensalism with humans remain understudied, effects currently attributed to natural phenomena may, in fact, be related to anthropogenic pressures on unmanaged populations of primates.

## Graphical abstract

Natural history collections are often thought to represent environments in a pristine natural state—free from human intervention—the so‐called “wild.” We found that among primates collected from their native ranges, 92% were from an environment that had some level of human impact, suggesting that the majority of presumed wild‐collected primate specimens may have lived in a human‐influenced environment during their lifetime. Effects attributed to natural phenomena may, in fact, be related to anthropogenic pressures on populations of primates, while unique effects related to ecological interactions with humans remain understudied.

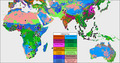

## 1 INTRODUCTION

The field of natural history has historically focused on the study and description of the Earth and its organisms, including their behaviors, ecological relationships, and evolution (Fleischner, 2005; Greene, 2005). The discipline of natural history grew substantially during periods of European imperialism in the 18th and 19th centuries, wherein Western naturalists traveled across colonized regions to describe and collect specimens in their natural settings (Greene, 2005). These early collectors specifically sought out geographic and ecological spaces devoid of human settlement and human impact (Denevan, 2011)—otherwise known as “the wild.” As such, historical zoological collections housed in natural history museums are generally thought to represent truly “wild” specimens.

**FIGURE 1:**
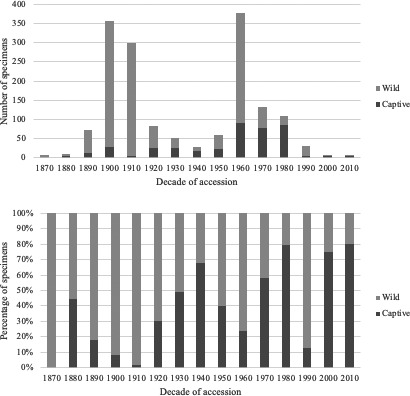
Wild and captive NMNH primate specimens by decade (1870–2010). Number (top) and percentage (bottom) of NMNH accessions of wild‐collected and captive specimens of nonhuman primates (*n* = 1632) by decade. More recent acquisitions show that “wild” specimens occupy a relatively smaller proportion of the total new accessions (bottom), while the overall number of new accessions reduces sharply (top)

However, many natural history specimens collected over the last century are animals who died in captive environments. These spaces, where humans and their built environments dominate, represent the ecological opposite of the wild and include zoos, sanctuaries, circuses, and biomedical facilities. For example, at the Smithsonian's National Museum of Natural History (NMNH), accessions of captive, nonhuman primate (NHP) specimens have grown since the 1960s (Figure 1). Furthermore, these captive specimens represent the majority of accessions in recent decades. While a large proportion of them were acquired from the Smithsonian's National Zoological Park (NZP), their provenance is often recorded as “locality unknown.” In these cases, the actual place of origin for the individual is recorded, but zoos and other captive environments are not considered the species’ place of origin and are therefore notated accordingly.

This clerical decision relates to a broader issue of how natural history specimens are differently treated and valued based on assumptions of human involvement. Researchers interested in evolution often avoid captive specimens based on the notion that human management interferes with natural processes (Delson et al., 2000; Fuentes, 2012). Evolutionary morphologists tend to exclude captive animals from their research because of potential anatomical distortions related to human control, particularly related to growth and development (Sigg et al., 1982). For example, the earliest observations of morphological differences between the skulls of wild lions (*Panthera leo*) and those obtained from the NZP (captive, though wild‐born) found the NZP skulls to be shorter, broader, and more massive than those of their wild conspecifics. Though Hollister (1917) attributed this to differences in prey capture and feeding behaviors, a follow‐up study using the same specimens emphasized the likely role pathology (e.g., osteitis deformans or Paget's disease) may have played in the appearance of the captive individuals (Howell, 1925). Similarly, O'Regan (2001) identified a significantly larger zygomatic breadth in a sample of captive lion and leopard (*P. pardus*) skulls compared with their wild conspecifics. In a morphometric study of captive and wild lion skulls, Zuccarelli (2004) demonstrated that most of the significant size and shape differences were in regions wherein the external stresses of mastication differentiated the populations, including palate length and width, mandibular length, and jaw height. Morphological differences in skulls were, again, reported for captive and wild lions and tigers (*P. tigris*), which were primarily attributed to drastically different diets (Hartstone‐Rose et al., 2014). Though neither O'Regan (2001) nor Zuccarelli (2004) indicated whether their captive specimens were wild or zoo‐born, Hartstone‐Rose et al. (2014) excluded any wild‐born specimens from their captive sample.

The wild versus captive dichotomy is becoming increasingly scrutinized across fields, with a growing interest in urban ecology and human–nonhuman species interfaces (Fuentes, 2012; Rivkin et al., 2019). Researchers have shown varied ways in which Indigenous and local communities have influenced the ecosystems in which they live, disproving the racist view by early European naturalists that non‐Europeans in colonized regions lived “within nature” without modifying it (e.g., the primitive and noble savage tropes) (Abrams & Nowacki, 2008; Denevan, 2011; Ellis et al., 2021; Piperno et al., 2021). Indeed, there is mounting evidence that humans across the globe have always shaped the surrounding nonhuman world and modified environments for millennia (Castree & Nash, 2006; Denevan, 2011; Ellis et al., 2021; Hofman & Rick, 2018; Ingold, 2004; Piperno et al., 2021; Stephens et al., 2019). This historical reality contradicts early naturalists’ underlying assumption that an organism's natural habitat was devoid of human impact, which is often considered a disturbance with negative influence on the environment. Rather, as part of many organisms’ natural ecosystems, humans both positively and negatively influence the behaviors and evolution of other species in a multitude of ways (Amano et al., 2021; Fuentes and Baynes‐Rock, 2017). Thus, a study of natural history should reflect the ways by which an organism's life adjusts to relationships with *all* other species in its ecosystem, including humans (Denevan, 2011). This suggests that the dichotomous categorization of natural history specimens as “wild” or “captive” ignores what is more accurately a spectrum of human influence, both historically and recently.

In primatology, NHPs within human‐modified environments and human interfaces have long been excluded from serious study in favor of those in naturalistic (“wild”) locations with less perceived human impact (Fuentes, 2012). However, with the emergence of the field of ethnoprimatology, there has been increasing recognition that human‐modified environments are ubiquitous and provide valuable knowledge about NHP behavior and evolution (Dore et al., 2017; Fuentes, 2012). Long histories of cohabitation, hunting pressures, and, in many cases, cultural significance suggest that NHPs have been adjusting to human influence for millennia (Amano et al., 2021); researchers incorporating this knowledge are now beginning to understand NHP responses to increasingly altered landscapes along a gradient (Hockings & McLennan, 2016), rather than a wild‐captive dichotomy as is commonly presented in museum records. Even more, the modern reality of anthropogenic climate change is that there are no environments that are untouched by human activities: They are all impacted by anthropogenic pressures to some degree, even those most distant from human reach (Allen et al., 2019; Jamieson et al., 2017). Thus, the designation of “wild” does not guarantee that an animal was born and lived free from the evolutionary pressures of chemicals, microbes, noises, lighting, and food that are associated with humans and built environments (Rivkin et al., 2019). Nonetheless, it is challenging to predict the biological impacts of different degrees of anthropogenic disturbance, especially through observational and field study (Loudon et al., 2014).

Large‐scale attempts are rarely made to understand the range of human‐modified environments represented by primate specimens in museum collections (however, see Amano et al., 2021). This may be due to the challenges of determining the precise provenance of many specimens, as well as the absence of a nondichotomous framework to assess the settings and conditions in which animals lived and died. These efforts are increasingly important, as studying anthropogenic influences on evolutionary processes is essential to understanding a rapidly changing natural world—and museum specimens are uniquely informative in this respect. NHP museum collections often span multiple centuries across many geographic regions and provide investigative insight into their anatomy and skeletal morphology that are inaccessible in living individuals. Knowledge of how NHPs respond to human‐induced habitat changes is not only of theoretical importance for examining the evolutionary flexibility of primates, but it is also fundamental for informing effective conservation management (Hockings & McLennan, 2016; Ontl, 2017).

To address these problems, we utilized anthrome maps created by Ellis et al. (2010) to identify and characterize the human‐influenced environments from which NHP museum specimens were collected. Anthromes, or anthropogenically modified biomes, are used in ecological research to describe the extent to which an ecosystem has been influenced or altered by anthropogenic changes to the land and environment (Ellis & Ramankutty, 2008). The mapped regions depict 19 anthrome types, based on human population and land‐use data. To investigate the applicability of these maps for museum collections research, we used them to plot the locations of origin for a large sample of NHP specimens collected by NMNH since the 19th century. The challenges and successes of this approach are discussed in a broader context of its utility for diverse collections and research questions in evolution and ecology.

## 2 MATERIALS AND METHODS

### 2.1 Sample

The study sample consisted of 875 specimens of nonhuman catarrhine primates (Table 1). These specimens are part of collections held by the Mammals Division of the Department of Vertebrate Zoology at NMNH, representing animals collected in 39 countries over more than 120 years (from 1882 to 2004). All study specimens were recorded as having been “wild‐collected” in NMNH accession records.

**TABLE 1:** Distribution of specimens in study sample (*N* = 875) by genus

Genus	Specimens (*n*)
*Gorilla*	71
*Pan*	26
*Pongo*	90
Subtotal	187
*Bunopithecus*	3
*Hylobates*	136
*Nomascus*	10
*Symphalangus*	8
Subtotal	157
*Allenopithecus*	1
*Cercopithecus*	65
*Chlorocebus*	136
*Erythrocebus*	16
*Macaca*	272
*Papio*	41
Subtotal	531
Total (*N*)	875

To determine the level of environmental anthropogenic impact among catarrhine primates, we included extant great apes, lesser apes, and cercopithecine monkeys in the sample; colobine primates, leaf‐eating monkeys, were not included due to COVID‐19 restrictions on access to NMNH collections since March 2020. Catarrhines are frugivorous/omnivorous, medium–large‐sized primates living in social groups, typically within arboreal or semiarboreal habitats (Rowe & Myers, 2016). Both cercopithecines and apes are ideal for examining anthropogenic influences because they exhibit an array of modern ecological success; some species are actively threatened with extinction due to human activities (e.g., all species of *Pongo, Pan,* and some species of *Cercopithecus*), while others are of least conservational concern (e.g., *Chlorocebus* and most species of *Macaca*; IUCN, 2019).

The sample contained 13 genera and 44 species, including 344 ape individuals (*Bunopithecus*, *Gorilla*, *Hylobates, Nomascus*, *Pan*, *Pongo*, and *Symphalangus*) and 531 cercopithecine monkey individuals (*Allenopithecus*, *Cercopithecus*, *Chlorocebus*, *Erythrocebus, Macaca*, and *Papio*). Using permanent molar eruption to assess the developmental age of each specimen (e.g., Smith et al., 1994), we determined that approximately 68% of the specimens were adults, 29% were juveniles, and 3% were infants. The proportion of males and females was slightly male‐biased (52.1% and 45.4%, respectively) with about 2.5% of unknown sex. Sex category was assigned for each specimen based on NMNH records and verified using skull and canine size dimorphism.

### 2.2 Methods

To reliably map our specimens in time and space, it was necessary to have the following information for each specimen in our sample: acquisition year, acquisition locality, and taxonomic designation. This information was compiled from NMNH online databases, specimen labels and containers, and accession records; all of these sources are publicly available, but not all have been digitized. Acquisition year and taxonomic designation were explicitly assigned by NMNH documentation; it is of note that acquisition dates may represent the date of field collection or the date of museum accession. An acquisition locality is an open‐ended variable that can be, for example, a forest, a county, an island, or a town; this is because geographic information is inconsistent among museum collections, especially over such a long period of time. Specimens with sufficient acquisition and locality information were plotted as points (hereafter “collection points”) in Google Maps (https://www.google.com/maps).

As precise geocoordinates are not available for many specimens, their provenance was determined on a case‐by‐case basis. Sufficient locality information to determine map location might include a named town, geocoordinates, a named natural preserve, or a named road/river with distances noted. In cases such as a named town, the point was placed within the town or on its immediate periphery. In the case of preserves, the collection point was placed in the approximate center, since anthropogenic activities are typically uniform within preserves. If the mapper (AE) could not confidently place a specimen given available records, then the specimen was removed from analysis. Although every attempt was made to accurately place collection points, there is some irreducible subjectivity in collection point placement, especially regarding historic specimens.

To determine levels of human influence on different habitats, we utilized the anthrome global biome maps created by Ellis et al. (2010). These publicly available maps delineate a range of terrestrial biomes based on human population density and land use, derived largely from archeological and ecological data sources. The authors classify different configurations of anthropogenic landscape changes around the globe at 5‐degree resolution, combining potential vegetation maps (Ramankutty & Foley, 1999) with anthrome maps (Ellis & Ramankutty, 2008) at century intervals from 1,700 to 2,000 using overlay analysis. The anthromes are classified into 19 distinct types and grouped into common land‐use schemas: Dense settlements, Villages, Croplands, Rangelands, Seminatural areas, and Wildlands (Table 2). Ellis et al. (2010) provide data for the 19th–21st centuries across the vast majority of known terrestrial biomes for anthrome type descriptions, including spatial data that are publicly available for download. For the present analysis, we used version 2 (https://ecotype.org/anthrome/v2) although newer datasets are now available (https://anthroecology.org/anthromes/maps).

**TABLE 2:** Description of fourteen anthrome types utilized in this study, adapted from Ellis et al. (2010)

Anthrome class	Anthrome type	Description
Dense settlements		Urban and other densely populated settlements
	Mixed settlements	Suburban settlements, townships, and rural settlements with high but fragmented human populations
Villages		Densely populated agricultural settlements
	Rice villages	Villages dominated by paddy rice
	Rainfed villages	Villages dominated by rainfed agriculture
Croplands		Lands used primarily for annual crops
	Residential rainfed croplands	Rainfed croplands with substantial human populations
	Populated rainfed croplands	Croplands with significant human populations; mixture of irrigated and rainfed crops
Rangelands		Lands used primarily for livestock grazing and pasture
	Residential rangelands	Rangelands, with substantial human populations
	Populated rangelands	Rangelands, with significant human populations
	Remote rangelands	Rangelands, without significant human populations
Seminatural lands		Inhabited lands, with minor use for permanent agriculture and settlements
	Residential woodlands	Forest regions with minor land use and with substantial populations
	Populated woodlands	Forest regions with minor land use and with significant populations
	Remote woodlands	Forest regions with minor land use and without significant populations
	Inhabited treeless and barren lands	Lands without natural tree cover, with only minor land use and a range of populations
Wildlands		Lands without human populations or substantial land use
	Wild woodlands	Forested regions and savannas
	Wild treeless and barren lands	Lands without natural tree cover (such as grasslands, shrublands, tundra, desert, and barren lands)

**FIGURE 2:**
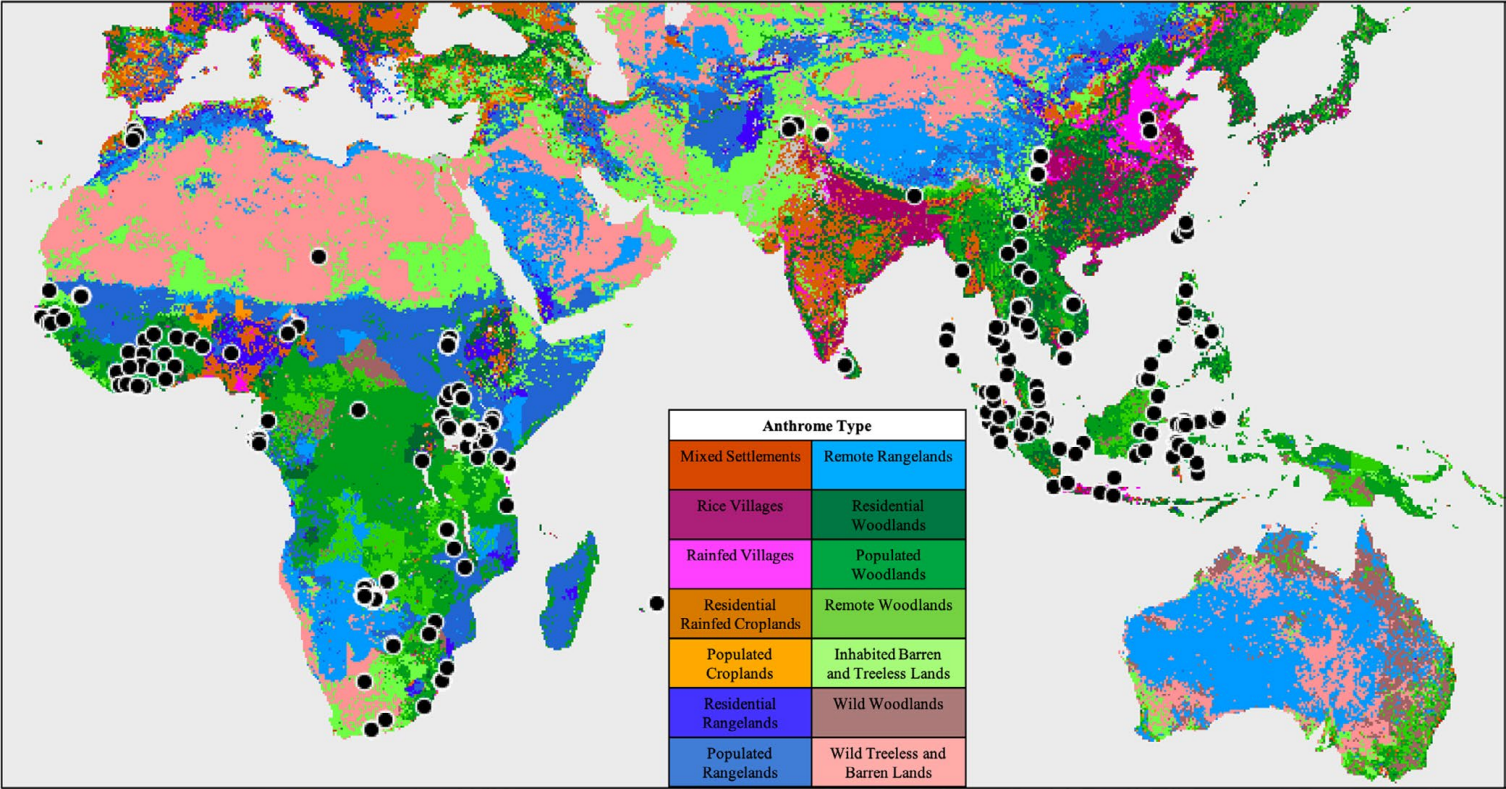
Sampled region anthrome map. 20th‐century map of anthrome types by Ellis et al. (2010) with collection points (black circles). The collection points correspond to locations where NHP specimens were collected, primarily in South‐East Asia, western Africa, and southern Africa

Using the software tools of QGIS (version 3.16, QGIS Development Team, 2021), we imported collection points for each specimen and anthrome map layers for each relevant century interval into a single map (Figure 2; full maps per century interval are available in Dryad doi: https://doi.org/10.5061/dryad.4f4qrfjcb). Based on the year of acquisition, primate specimens were grouped into the 19th‐, 20th‐, or 21st‐century anthrome map. From QGIS, we then exported the specimen and corresponding anthrome data into MS Excel (2021, v. 16.47.1) for analysis.

## 3 RESULTS

**FIGURE 3:**
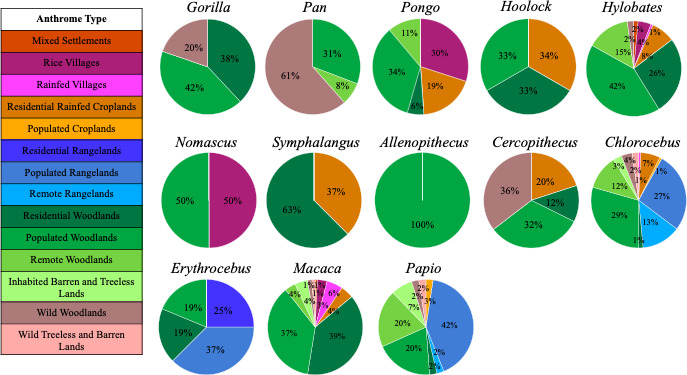
Distribution of anthromes by genus. Anthrome type composition of wild‐collected specimens for each of the thirteen nonhuman primate genera in study sample (*N* = 875)

**FIGURE 4:**
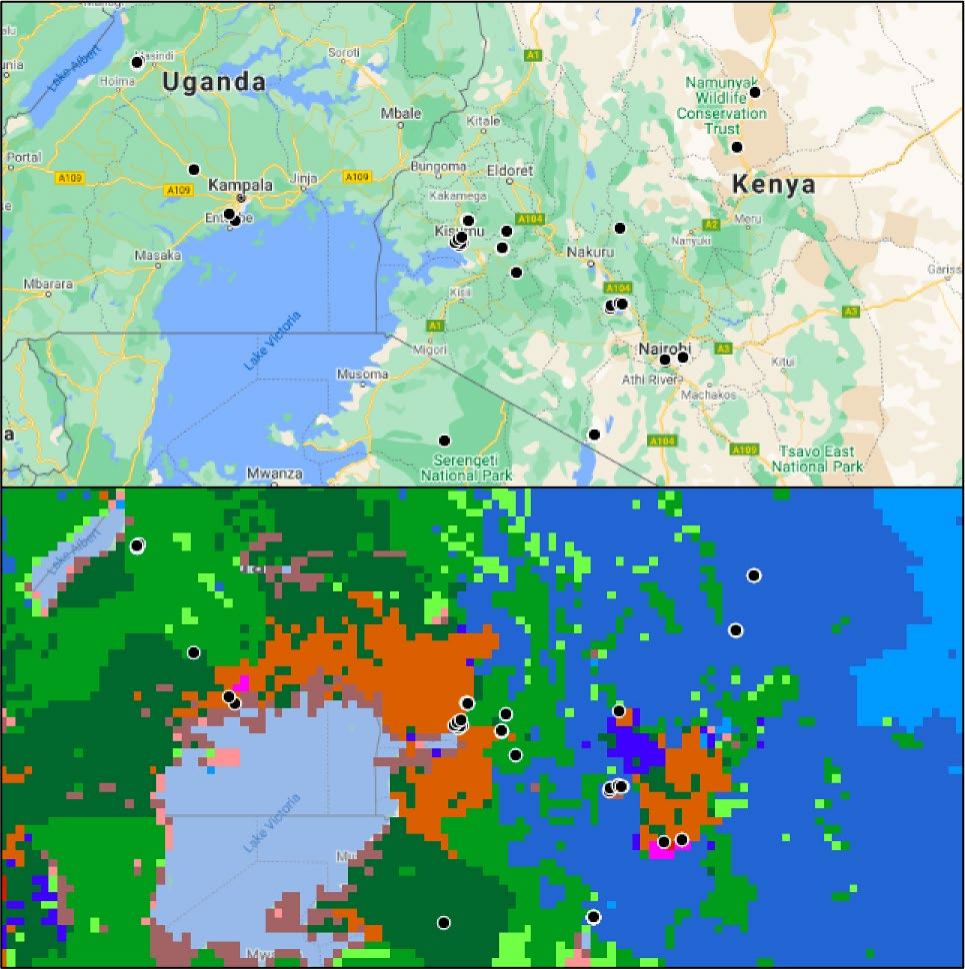
Selected anthrome map, Lake Victoria Region. Lake Victoria anthromes and political boundaries, 20th century. These images depict Lake Victoria (shown in light blue) and surrounding countries Kenya, Uganda, Rwanda, and Tanzania. The black dots represent collection points of primates in this study. The top image shows the area with political boundaries and roads in modern‐day, and the bottom image depicts the same area with the 20th c. anthrome layer added. The bottom image highlights the granularity of anthrome data at the regional level. Anthrome type colors are defined in Figures 2 and 3

The plotting of the collection points for each NHP specimen on anthrome maps showed that these specimens were collected from a wide range of anthrome types (see Figures 2, 3, 4). Of the 19 anthrome types classified by Ellis et al. (2010), primates from the NMNH collections were plotted within 14 of them (Table 2, Figures 2 and 3). The primary anthrome group occupied by primates in our study were seminatural lands (66.6%, Table 3). These lands are defined as inhabited regions with only minor utilization of resources for settlements or agriculture (Table 2) and include inhabited woodlands and uninhabited/ uninhabitable, barren lands.

**TABLE 3:** Distribution of specimens by anthrome

Anthrome group	*n*	% of Total	Anthrome type	*n*
Dense settlements	4	0.46		
			Mixed settlements	4
Villages	64	7.31		
			Rice villages	47
			Rainfed villages	17
Croplands	68	7.77		
			Residential rainfed croplands	66
			Populated rainfed croplands	2
Rangelands	83	9.49		
			Residential rangelands	4
			Populated rangelands	60
			Remote rangelands	19
Seminatural	583	66.63		
			Residential woodlands	193
			Populated woodlands	305
			Remote woodlands	66
			Inhabited treeless and barren lands	19
Wildlands	73	8.34		
			Wild woodlands	66
			Wild treeless and barren lands	7
Total	875	100.00	Total	875

Primates occupy a wide range of terrestrial biomes, as illustrated by the anthrome results. These taxa, however, are not represented equally among human‐modified environmental types. For example, Figure 3 illustrates the proportion contributed by each anthrome type to the total sample per genera. While some genera (*Chlorocebus*, *Hylobates*, and *Macaca*) were collected from a wide variety of anthrome types, others (*Gorilla*, *Nomascus*, and *Symphalangus*) showed limited anthrome diversity. The most common anthrome types, across genera, were Populated Woodlands (34.8% of total sample) and Residential Woodlands (22.0%); both types are included in the anthrome group Seminatural (Table 2).

While we expected some variation in anthrome types from which the primates in our sample were collected, the amount of anthropogenic influence is surprisingly high; 91.7% of our sample is derived from some type of human‐influenced landscape, whereas only 8.3% come from Wildlands (regions without substantial human populations or land use). While the vast majority of our sample comes from Seminatural areas, a further 17.2% derived from Croplands and Rangelands, and 7.8% come from Villages or Dense settlements. The number of individuals found in each anthrome type, and the corresponding percentage of the total each anthrome represents, is listed in Table 3.

## 4 DISCUSSION

### 4.1 Environmental variation across time

The most recent chapter of the NMNH primate collection is distinct in its composition from the earliest period. Over the roughly 120 years of collecting, there has been a marked decline in wild specimen accession rates (Figure 1). The more recent reduction in wild‐collected primates is likely due to designated conservation statuses and habitat loss (Gordon et al., 2013), alongside increased regulation of animal care and use in research (see Sikes, 2016; Sikes et al., 2019). Thus, new acquisitions are reduced overall and increasingly represent individuals from human‐managed, or human‐impacted, environments. This trend is apparent within the simple wild‐captive dichotomy and becomes clearer with the level of human influence on environments illustrated among the supposed wild‐collected specimens (Figure 3).

Specimens collected from Smithsonian‐sponsored expeditions (e.g., the Smithsonian‐Roosevelt East African Expedition from 1909 to 1910, see Sterling, 2005) are individuals who lived within an environment that was considered free from human intervention, as it was preferred to study an organism from its natural, “wild” habitat. To our knowledge, all of the wild‐collected individuals in our study were collected under a common mission to understand the natural world. However, this perspective minimizes the role of land‐use changes, local residents, and travel limitations by collection scientists, all of which contribute to the reality of anthropogenic influences among museum collections (for sampling biases due to access routes, see Oliveira et al., 2016). For example, Figure 4 shows two maps of Lake Victoria and the surrounding region, with collections points of specimens collected between 1900 and 1999. These maps depict the political boundaries and roads of modern‐day Kenya, Uganda, Rwanda, and Tanzania, and the anthromes which correspond to the region from the 20th century. Together, these images offer an illustration of specimen collection patterns as scientists followed roads, focused efforts in forested refuges, and collected on the outskirts of towns. The granularity of anthrome data is also visible, where varying biome types abut one another in short succession, particularly around populated areas.

While Figure 4 offers a granular view of one region, anthropogenic influences have changed dramatically over the last 100 years and this map cannot expound on that detail. One limitation of this study is that until very recently, anthrome maps were only in centennial slices of time (see Ellis et al., 2021 for updated anthrome datasets). Especially during the 20th century, human populations grew rapidly, from around 1.6 billion in 1900 to 6.1 billion in 2000 (population estimates from Worldometer: https://www.worldometers.info/world‐population/world‐population‐by‐year/). Furthermore, human land use changed significantly on a decadal scale: between 1980 and 2000, cropland area increased by ∼50% in East Africa and by ∼25% in West Africa, and nearly 60% of new agricultural land across the continent came from intact forests (Gibbs et al., 2010). Therefore, it is likely that our study may be underestimating the amount of anthropogenic impact during this time period.

### 4.2 Environmental variation across taxa

The taxonomic patterns across human‐modified environments revealed by the present analysis are largely explained by the ecological variation among nonhuman primates. Extant hominoids, including greater and lesser apes, typically experience population decline when in close contact with humans (Walsh et al., 2003), whereas a number of cercopithecine monkeys exhibit documented synanthropy, especially *Macaca*, *Papio*, and *Chlorocebus* (Engel et al., 2010; Klegarth, 2016; Knauf & Jones‐Engel, 2020; Nyamota et al., 2018).

Historical disregard of anthropogenic environmental pressures reduces the ability of researchers to understand a potentially adaptive trend in catarrhine primates, especially monkeys. Some monkeys can successfully live synanthropically, likely doing so because of access to human foods. Several species included in this study are known to crop‐raid (e.g., *Chlorocebus sabaeus*, Dore, 2018; *Papio anubis*, Alberts & Altmann, 2006; *Pan troglodytes*, Hockings et al., 2012), and/or live near human settlements on a consistent basis (e.g., *Macaca* sp., Fuentes & Wolfe, 2002). Richard et al. (1989) argued that human affiliative behaviors are so important to macaque evolutionary success that the adaptive suite created by these acclimatizations may actually drive speciation and explain existing species diversity. More recent work has highlighted these synanthropic species as sentinels or vectors of emerging infectious disease among humans (see Knauf & Jones‐Engel, 2020 for review).

In the present study, we identified specimens of two genera from Mixed settlements (Figure 3), an anthrome type defined as “Suburbs, towns and rural settlements with high but fragmented populations” (Ellis et al., 2010:591). Identified specimens were *Hylobates*, the most geographically diverse genus among gibbons, and *Macaca*, the second most geographically diverse genus of any primate ever to inhabit the planet (Fooden, 2000; Maestripieri, 2008). There is arguably an ecological advantage for some primate populations if they can maintain adequate habitat while coexisting with human populations. Direct consideration of the anthropogenic impact of an animal's native habitat, and not only as a destructive force, provides insight into the adaptive strategies of free‐roaming animals living in any anthrome.

This flexibility within primate taxa has allowed for some successful acclimatizations, including synanthropic lifeways (McLennan et al., 2017), although the majority of primates species have seen drastic reductions in their preferred woodland habitats over the last century (Cowlishaw, 1999; Estrada et al., 2017). Many primate species prefer predominantly arboreal habitats, especially apes (Rowe & Myers, 2016), yet some monkey species prefer a more terrestrial landscape (e.g., *Erythrocebus patas;* Gron, 2006). Thus, deforestation is the number one threat for most endangered primate species, due to agricultural demands and wood‐harvesting industries (Estrada et al., 2017; IUCN, 2019). Primates with thriving populations are those utilizing a variety of anthromes and often exist in close proximity to human populations (McLennan et al., 2017; see Nijman and Nekaris (2010) for human attitudes about these changes in Sri Lankan monkeys).

Primate home ranges, like those of many other animals, vary by species, body size, diet, and anthropogenic influence (Clutton‐Brock, 2012; Jaman & Huffman, 2013). Generally, terrestrial species have larger ranges than arboreal species, because arboreal territory is defined in three dimensions (Carbone et al., 2005; Pearce et al., 2013). Among macaques, forested ranges can exceed 15 km^2^ (Lindberg, 1971), while urbanized ranges may be as small as 0.01‐3km^2^ due to an abundance of readily available anthropogenic foods within a smaller range (Seth et al., 1989). In this study, specimen collection locales represent a single moment in time and therefore can only offer a snapshot of anthromes occupied by primates within their native ranges. This method cannot provide information about the various biomes included within an individual's home range; for primates, this almost certainly means we are underestimating the variety of anthromes utilized by any given population.

Future studies would benefit from expanded taxonomic applications, within the order Primates and beyond. Colobine monkeys were not included in this study, but it is likely that this group would also display diversity in anthrome occupation. For example, *Colobus* sp. tend not to fare well in the presence of human populations (Siobhan Cooke, *personal communication*), while *Semnopithecus* sp. are commonly found in human settlements (Chauhan & Pirta, 2010; Chhangani & Mohnot, 2006; Koenig & Borries, 2001). Colobines are more folivorous and arboreal than cercopithecine monkeys, generally, and these adaptations likely affect the ways in which these monkeys interact with human communities.

### 4.3 Applications for ecological and evolutionary research

Existing natural history collections already contain specimens from areas with documented anthropogenic changes, but this is not often considered when specimens are used for research. Arguably, this is largely due to the widespread use of the wild‐captive dichotomy when characterizing specimens by the level of anthropogenic impact on their environments. Captive primates are (usually) easy to identify from museum records, whereas more nuanced data for noncaptive primates are almost invisible to detect without a specific attempt to categorize them as such. As the present study demonstrates, many specimens within museum collections typically are not from the “wild” in the implicit sense, or even from Wildlands (see Table 3 and Figure 3) in the categorized sense. Though many of the animals in this study may have been unmanaged by humans or may have had limited human contact during their lifetime, most are likely to have endured human populations and land usage changes within their native biomes. Additionally, a large portion of these specimens are macaques from South‐East Asia (80% of macaque sample, 25% of the total sample), where people regularly provision nonhuman primates with food consistent with centuries of cultural and religious significance (Fuentes, 2010; Peterson et al., 2015; Radhakrishna, 2018; Riley & Priston, 2010).

It is likely that some attributes of primate morphology, ecology, or behavior, which had been previously attributed to a natural condition, are in fact due to human‐introduced factors. For example, the captive environment (e.g., enclosure complexity) has been shown to affect the ontogenetic trajectory of long bone cross‐sectional diaphyses in captive gorillas (*Gorilla gorilla*) compared with their wild conspecifics, a result attributed to a decreased climbing frequency and/upon non‐natural substrates (Canington et al., 2018). Some heavily human‐impacted spaces, such as captive institutions, alter the bodies of nonhuman primates in unexpected ways. As one example, recent studies have indicated that some macaque populations may be experiencing morphological changes in ankle shape due to substrate exposure (Turley & Frost, 2018; Turley et al., 2015). This work offers evidence that macaques who live the majority of their lifetime on flat, hard substrates will tend to feature smaller tibio‐talar articulation surface and less flexibility which is more conducive to terrestrial locomotion, whereas the same species living in a more varied substrate environment (such as an arboreal environment) will feature a broader articulation surface and increased flexibility more conducive to arboreal movement. Tibio‐talar articulation morphology has been long assumed to be static, indicating preexisting adapted locomotor patterns, rather than a use‐based characteristic (see Simons et al., 2019; Turley & Frost, 2018). Further, applications of this method are showing intriguing results in human populations from varying environments (see Sorrentino et al., 2020). Not only does this research highlight the developmentally plastic nature of skeletal elements, but also that human‐impacted environments may literally shape future generations of these species.

Reconstructing anthropogenic influences (based on global anthrome data, or perhaps based on existing, ethnographic museum records) encourages new ways to interpret and utilize museum specimens. Detailed knowledge of the contemporaneous anthropogenic impacts on the environment can illuminate relationships between anthrome type and nonhuman behavior, morphology, and health outcomes among existing collections (Donihue & Lambert, 2015; Loudon et al., 2006, 2014). Ultimately, future studies utilizing museum specimens would greatly benefit from a dataset integrating their taxon or geographic area of study, with historical documentation of the environment research and ethnoprimatological data reflecting animal behaviors and customs. In concert with modern technologies, this approach allows novel investigations involving biomolecular work, morphometrics, and urban ecologies.

## 5 CONCLUSION

Our findings suggest that the majority of nonhuman catarrhine primate specimens in NMNH collections are not from the “wild.” Among the 1632 specimens surveyed for this study, 1,220 (74.7%) were collected from their native habitats, and among these, 875 specimens included enough information to assign them to an anthrome based on original collection date and mapped locale. We show that 91.7% of mapped specimens were collected from a human‐impacted landscape, meaning that only 73 individuals (8.3% of mapped specimens, or 4.5% of all surveyed specimens) were demonstrably from a habitat with little to no direct human impacts.

Museum specimens, collected from native habitats or human‐managed institutions, may be better understood on a scale of anthropogenic influences on the environments from which they originate, rather than a wild‐captive dichotomy that oversimplifies the ecological and biological reality of their lives. There is far more environmental variation to be investigated with respect to human influence than previously recognized in these collections (e.g., Tomiya & Meachen, 2018). Additionally, these patterns could be extended further into the past, when new anthrome datasets spanning the last 12,000 years of human land‐use change (Ellis et al., 2021) are integrated with zooarcheological or subfossil collections. In fact, this approach can be useful for more comprehensive niche modeling efforts that incorporate human land‐use variables, exploring historical species ranges and the relationship between Indigenous communities and wildlife, as well as conservation efforts that integrate long‐term historical–ecological data. The accuracy and accessibility of relevant provenance data used for the specimen assignments are critical as these collections continue to grow. With these efforts, further research can increase our understanding of how human‐impacted environments, through the lens of evolutionary biology, create challenges, evoke responses, and reveal connections between humans and other animals across many biomes worldwide (for a discussion of natural history collection as understudied sources of evolutionary biology research, see Holmes et al., 2016).

Incorporating anthropogenic environmental information into research on nonhuman primates and other zoological specimens is a crucial next step to more fully comprehend human impacts on the environment, past and present. Natural history museums, and the researchers utilizing their collections, must continue to acknowledge the influences of humans on their specimens to increase our understanding of the anthropogenic impacts on animal bodies and behaviors. Some nonhuman primates, and other organisms, have exhibited remarkable resilience and adaptation in the face of anthropogenic pressures, while many others have suffered steep declines or been eradicated altogether (Estrada et al., 2017). By recognizing the various ways that humans articulate with and alter their environments, we can understand more fully how these pressures affect other aspects of biology such as development, the microbiome, disease ecology, and morphology.

## CONFLICT OF INTEREST

We have no competing interests to declare.

## AUTHOR CONTRIBUTION

**Andrea R. Eller:** Conceptualization (lead); Data curation (lead); Formal analysis (equal); Methodology (equal); Visualization (lead); Writing‐original draft (equal); Writing‐review & editing (supporting). **Stephanie L. Canington:** Conceptualization (supporting); Data curation (equal); Methodology (equal); Writing‐review & editing (equal). **Sana T. Saiyed:** Data curation (equal); Formal analysis (supporting); Methodology (equal); Software (lead); Visualization (equal); Writing‐review & editing (equal). **Rita M. Austin:** Conceptualization (supporting); Writing‐review & editing (equal). **Courtney A. Hofman:** Conceptualization (supporting); Formal analysis (supporting); Methodology (equal); Supervision (equal); Writing‐review & editing (supporting). **Sabrina B. Sholts:** Conceptualization (supporting); Methodology (supporting); Supervision (equal); Writing‐original draft (equal); Writing‐review & editing (equal).

## Data Availability

Sample dataset, specimen, and locality information, MS Excel spreadsheet (.xlsx): Dryad https://doi.org/10.5061/dryad.4f4qrfjcb. Collection point map layers by century (1800, 1900, 2000), QGIS map layers (.qgz), zip file: Dryad https://doi.org/10.5061/dryad.4f4qrfjcb. Images of complete maps by century (1800, 1900, 2000), PDF (.pdf): Dryad https://doi.org/10.5061/dryad.4f4qrfjcb

## References

[ece38006-bib-0001] Abrams, M. D., & Nowacki, G. J. (2008). Native Americans as active and passive promoters of mast and fruit trees in the eastern USA. The Holocene, 18(7), 1123–1137. 10.1177/0959683608095581

[ece38006-bib-0002] Alberts, S. C., & Altmann, J. (2006). The evolutionary past and the research future: Environmental variation and life history flexibility in a primate lineage. In L. Swedell & S. R. Leigh (Eds.), Reproduction and fitness in baboons: Behavioral, ecological, and life history perspectives (pp. 277–303). Springer Science & Business Media.

[ece38006-bib-0003] Allen, S., Allen, D., Phoenix, V. R., Le Roux, G., Jiménez, P. D., Simonneau, A., Binet, S., & Galop, D. (2019). Atmospheric transport and deposition of microplastics in a remote mountain catchment. Nature Geoscience, 12(5), 339–344. 10.1038/s41561-019-0335-5

[ece38006-bib-0004] Amano, N., Wang, Y. V., Boivin, N., & Roberts, P. (2021). ‘Emptying Forests?’ Conservation implications of past human‐primate interactions. Trends in Ecology & Evolution, 36(4), 345–359. 10.1016/j.tree.2020.12.004 33431163

[ece38006-bib-0005] Canington, S. L., Sylvester, A. D., Burgess, M. L., Junno, J. A., & Ruff, C. B. (2018). Long bone diaphyseal shape follows different ontogenetic trajectories in captive and wild gorillas. American Journal of Physical Anthropology, 167(2), 366–376. 10.1002/ajpa.23636 30159891

[ece38006-bib-0006] Carbone, C., Cowlishaw, G., Isaac, N. J. B., & Rowcliffe, J. M. (2005). How far do animals go? Determinants of day range in mammals. American Naturalist., 165, 290–297. 10.1086/426790 15729658

[ece38006-bib-0007] Castree, N., & Nash, C. (2006). Posthuman geographies. Social & Cultural Geography, 7(4), 501–504. 10.1080/14649360600825620

[ece38006-bib-0008] Chauhan, A., & Pirta, R. S. (2010). Socio‐ecology of two species of nonhuman primates, rhesus monkey (*Macaca mulatta*) and Hanuman langur (*Semnopithecus entellus*), in Shimla, Himachal Pradesh. Journal of Human Ecology, 30(3), 171–177.

[ece38006-bib-0009] Chhangani, A. K., & Mohnot, S. M. (2006). Ranging behaviour of Hanuman langurs (*Semnopithecus entellus*) in three different habitats. Primate Conservation, 2006(21), 171–177. 10.1896/0898-6207.21.1.171

[ece38006-bib-0010] Clutton‐Brock, T. H. (Ed.) (2012). Primate ecology: studies of feeding and ranging behavior in lemurs, monkey and apes. Elsevier.

[ece38006-bib-0011] Cowlishaw, G. (1999). Predicting the pattern of decline of African primate diversity: An extinction debt from historical deforestation. Conservation Biology, 13(5), 1183–1193. 10.1046/j.1523-1739.1999.98433.x

[ece38006-bib-0012] Delson, E., Terranova, C. J., Jungers, W. L., Sargis, E. J., & Jablonski, N. G. (2000). Body mass in Cercopithecidae (Primates, Mammalia): estimation and scaling in extinct and extant taxa. Anthropological papers of the AMNH; no. 83.

[ece38006-bib-0013] Denevan, W. M. (2011). The “pristine myth” revisited. Geographical Review, 101(4), 576–591. 10.1111/j.1931-0846.2011.00118.x

[ece38006-bib-0014] Donihue, C. M., & Lambert, M. R. (2015). Adaptive evolution in urban ecosystems. Ambio, 44(3), 194–203. 10.1007/s13280-014-0547-2 25056615 PMC4357625

[ece38006-bib-0015] Dore, K. M. (2018). Ethnoprimatology without conservation: The political ecology of farmer–green monkey (Chlorocebus sabaeus) relations in St. Kitts, West Indies. International Journal of Primatology, 39(5), 918–944. 10.1007/s10764-018-0043-9

[ece38006-bib-0016] Dore, K. M., Riley, E., & Fuentes, A. (Eds.) (2017). Ethnoprimatology, Vol. 76. Cambridge University Press.

[ece38006-bib-0017] Ellis, E. C., Gauthier, N., Goldewijk, K. K., Bird, R. B., Boivin, N., Díaz, S., Fuller, D. Q., Gill, J. L., Kaplan, J. O., Kingston, N., Locke, H., McMichael, C. N. H., Ranco, D., Rick, T. C., Shaw, M. R., Stephens, L., Svenning, J.‐C., & Watson, J. E. M. (2021). People have shaped most of terrestrial nature for at least 12,000 years. Proceedings of the National Academy of Sciences, 118(17), e2023483118. 10.1073/pnas.2023483118 PMC8092386 33875599

[ece38006-bib-0018] Ellis, E. C., Klein Goldewijk, K., Siebert, S., Lightman, D., & Ramankutty, N. (2010). Anthropogenic transformation of the biomes, 1700 to 2000. Global Ecology and Biogeography, 19(5), 589–606. 10.1111/j.1466-8238.2010.00540.x

[ece38006-bib-0019] Ellis, E. C., & Ramankutty, N. (2008). Putting people in the map: Anthropogenic biomes of the world. Frontiers in Ecology and the Environment, 6, 439–447. 10.1890/070062

[ece38006-bib-0020] Engel, G., O'Hara, T. M., Cardona‐Marek, T., Heidrich, J., Chalise, M. K., Kyes, R., & Jones‐Engel, L. (2010). Synanthropic primates in Asia: Potential sentinels for environmental toxins. American Journal of Physical Anthropology, 142(3), 453–460. 10.1002/ajpa.21247 20033917 PMC2901096

[ece38006-bib-0021] Estrada, A., Garber, P. A., Rylands, A. B., Roos, C., Fernandez‐Duque, E., Di Fiore, A., Nekaris, K.‐I., Nijman, V., Heymann, E. W., Lambert, J. E., Rovero, F., Barelli, C., Setchell, J. M., Gillespie, T. R., Mittermeier, R. A., Arregoitia, L. V., de Guinea, M., Gouveia, S., Dobrovolski, R., … Li, B. (2017). Impending extinction crisis of the world’s primates: Why primates matter. Science Advances, 3(1), e1600946. 10.1126/sciadv.1600946 28116351 PMC5242557

[ece38006-bib-0022] Fleischner, T. L. (2005). Natural history and the deep roots of resource management. Natural Resources Journal, 45(1), 1–13.

[ece38006-bib-0023] Fooden, J. (2000). Systematic review of rhesus macaque, *Macaca mulatta* (Zimmermann, 1780). Fieldiana Zool, 96, 1–180.

[ece38006-bib-0024] Fuentes, A. (2010). Naturalcultural encounters in Bali: Monkeys, temples, tourists, and ethnoprimatology. Cultural Anthropology, 25(4), 600–624. 10.1111/j.1548-1360.2010.01071.x

[ece38006-bib-0025] Fuentes, A. (2012). Ethnoprimatology and the anthropology of the human‐primate interface. Annual Review of Anthropology, 41, 101–117. 10.1146/annurev-anthro-092611-145808

[ece38006-bib-0100] Fuentes, A., & Baynes‐Rock, M. (2017). Anthropogenic landscapes, human action and the process of co‐construction with other species: Making anthromes in the Anthropocene. Land, 6(1), 15. 10.3390/land6010015

[ece38006-bib-0026] Fuentes, A., & Wolfe, L. D. (Eds.) (2002). Primates face to face: The conservation implications of human‐nonhuman primate interconnections, Vol. 29. Cambridge University Press.

[ece38006-bib-0027] Gibbs, H. K., Ruesch, A. S., Achard, F., Clayton, M. K., Holmgren, P., Ramankutty, N., & Foley, J. A. (2010). Tropical forests were the primary sources of new agricultural land in the 1980s and 1990s. Proceedings of the National Academy of Sciences, 107(38), 16732–16737. 10.1073/pnas.0910275107 PMC2944736 20807750

[ece38006-bib-0028] Gordon, A. D., Marcus, E., & Wood, B. (2013). Great ape skeletal collections: Making the most of scarce and irreplaceable resources in the digital age. American Journal of Physical Anthropology, 152, 2–32. 10.1002/ajpa.22391 24249590

[ece38006-bib-0029] Greene, H. W. (2005). Organisms in nature as a central focus for biology. Trends in Ecology & Evolution, 20(1), 23–27. 10.1016/j.tree.2004.11.005 16701336

[ece38006-bib-0030] Gron, K. J. (2006). Primate Factsheets: Patas monkey (*Erythrocebus patas*) Behavior. http://pin.primate.wisc.edu/factsheets/entry/patas_monkey/behav

[ece38006-bib-0031] Hartstone‐Rose, A., Selvey, H., Villari, J. R., Atwell, M., & Schmidt, T. (2014). The three‐dimensional morphological effects of captivity. PLoS One, 9(11), e113437. 10.1371/journal.pone.0113437 25409498 PMC4237414

[ece38006-bib-0032] Hockings, K. J., Anderson, J. R., & Matsuzawa, T. (2012). Socioecological adaptations by chimpanzees, Pan troglodytes verus, inhabiting an anthropogenically impacted habitat. Animal Behaviour, 83(3), 801–810. 10.1016/j.anbehav.2012.01.002

[ece38006-bib-0033] Hockings, K. J., & McLennan, M. R. (2016). Problematic primate behaviour in agricultural landscapes: Chimpanzees as ‘pests’ and ‘predators’. In M. Waller (Ed.), Ethnoprimatology. Developments in primatology: Progress and prospects (pp. 137–156). Springer. 10.1007/978-3-319-30469-4_8

[ece38006-bib-0034] Hofman, C. A., & Rick, T. C. (2018). Ancient Biological Invasions and Island Ecosystems: Tracking Translocations of Wild Plants and Animals. Journal of Archaeological Research, 26, 65–115. 10.1007/s10814-017-9105-3

[ece38006-bib-0035] Hollister, N. (1917). Some effects of environment and habit on captive lions. Proceedings of the United States National Museum, 53(2196), 177–193. 10.5479/si.00963801.53-2196.177

[ece38006-bib-0036] Holmes, M. W., Hammond, T. T., Wogan, G. O., Walsh, R. E., LaBarbera, K., Wommack, E. A., Martins, F. M., Crawford, J. C., Mack, K. L., Bloch, L. M., & Nachman, M. W. (2016). Natural history collections as windows on evolutionary processes. Molecular Ecology, 25(4), 864–881. 10.1111/mec.13529 26757135 PMC4755843

[ece38006-bib-0037] Howell, A. B. (1925). Pathologic skulls of captive lions. Journal of Mammalogy, 6(3), 163–168. 10.2307/1373626

[ece38006-bib-0038] Ingold, T. (2004). Beyond biology and culture. The meaning of evolution in a relational world. Social Anthropology, 12(2), 209–221. 10.1017/S0964028204000291

[ece38006-bib-0039] IUCN: International Union for Conservation of Nature (2019). The IUCN Red List of Threatened Species. Version 2018‐2. http://www.iucnredlist.org

[ece38006-bib-0040] Jaman, M. F., & Huffman, M. A. (2013). The effect of urban and rural habitats and resource type on activity budgets of commensal rhesus macaques (*Macaca mulatta*) in Bangladesh. Primates, 54(1), 49–59. 10.1007/s10329-012-0330-6 22987063

[ece38006-bib-0041] Jamieson, A. J., Malkocs, T., Piertney, S. B., Fujii, T., & Zhang, Z. (2017). Bioaccumulation of persistent organic pollutants in the deepest ocean fauna. Nature Ecology & Evolution, 1(3), 1–4. 10.1038/s41559-016-0051 28812719

[ece38006-bib-0042] Klegarth, A. R. (2016). Synanthropy. The International Encyclopedia of Primatology, 1, 1–5.

[ece38006-bib-0043] Knauf, S., & Jones‐Engel, L. (2020). An introduction to one health and neglected diseases in monkeys. In Neglected Diseases in Monkeys (pp. 1–5). Springer.

[ece38006-bib-0044] Koenig, A., & Borries, C. (2001). Socioecology of Hanuman langurs: The story of their success. Evolutionary Anthropology: Issues, News, and Reviews: Issues, News, and Reviews, 10(4), 122–137. 10.1002/evan.1026

[ece38006-bib-0045] Lindberg, D. G. (1971). The rhesus monkey in Northern India: an ecological and behavioral study. In L. A. Rosenblum (Ed.), Primate Behavior (Vol. 2, pp. 1‐106). Academic Press.

[ece38006-bib-0046] Loudon, J. E., Grobler, J. P., Sponheimer, M., Moyer, K., Lorenz, J. G., & Turner, T. R. (2014). Using the stable carbon and nitrogen isotope compositions of vervet monkeys (*Chlorocebus pygerythrus*) to examine questions in ethnoprimatology. PLoS One, 9(7), e100758. 10.1371/journal.pone.0100758 25010211 PMC4091945

[ece38006-bib-0047] Loudon, J. E., Howells, M. E., Fuentes, A. (2006). The importance of integrative anthropology: A preliminary investigation employing primatological and cultural anthropological data collection methods in assessing human‐monkey co‐existence in Bali, Indonesia. Ecological and Environmental Anthropology (University of Georgia), 26. https://digitalcommons.unl.edu/icwdmeea/26

[ece38006-bib-0048] Maestripieri, D. (2008). Macachiavellian intelligence: How rhesus macaques and humans have conquered the world. University of Chicago Press.

[ece38006-bib-0049] McLennan, M. R., Spagnoletti, N., & Hockings, K. J. (2017). The implications of primate behavioral flexibility for sustainable human‐primate coexistence in anthropogenic habitats. International Journal of Primatology, 38, 105–121. 10.1007/s10764-017-9962-0

[ece38006-bib-0050] Nijman, V., & Nekaris, K. A. I. (2010). Effects of deforestation on attitudes and levels of tolerance towards commensal primates (Cercopithecidae) in Sri Lanka. International Journal of Pest Management, 56(2), 153–158. 10.1080/09670870903248850

[ece38006-bib-0051] Nyamota, R., Vincent, O., Edwin, M., Moses, O., Jandouwe, V., & Maamun, J. (2018). Extensive diversity of SIVagm infecting synanthropic African green monkeys and Olive Baboons in Kenya. International Journal of Infectious Diseases, 73, 85. 10.1016/j.ijid.2018.04.3618 29913285

[ece38006-bib-0052] Oliveira, U., Paglia, A. P., Brescovit, A. D., de Carvalho, C. J., Silva, D. P., Rezende, D. T., Leite, F. S. F., Batista, J. A. N., Barbosa, J. P. P. P., Stehmann, J. R., & Ascher, J. S. (2016). The strong influence of collection bias on biodiversity knowledge shortfalls of Brazilian terrestrial biodiversity. Diversity and Distributions, 22(12), 1232–1244.

[ece38006-bib-0053] Ontl, K. M. B. (2017). Chimpanzees in the Island Of Gold: Impacts of artisanal small‐scale gold mining on chimpanzees (Pan troglodytes verus) in Fongoli, Senegal. Dissertation. Iowa State University.

[ece38006-bib-0054] O'Regan, H. J. (2001). Morphological effects of captivity in big cat skulls. In S. Wehnelt & C. Hudson (Eds.), Proceedings of the 3rd Annual Symposium on Zoo Research, Chester Zoo, Chester, UK, 9‐10th July 2001 (pp. 18–22). The North of England Zoological Society.

[ece38006-bib-0055] Pearce, F., Carbone, C., Cowlishaw, G., & Isaac, N. J. (2013). Space‐use scaling and home range overlap in primates. Proceedings of the Royal Society B: Biological Sciences, 280(1751), 20122122. 10.1098/rspb.2012.2122 PMC3574404 23193124

[ece38006-bib-0056] Peterson, J. V., Riley, E. P., & Putu Oka, N. (2015). Macaques and the ritual production of sacredness among Balinese transmigrants in South Sulawesi. Indonesia. American Anthropologist, 117(1), 71–85. 10.1111/aman.12166

[ece38006-bib-0057] Piperno, D. R., McMichael, C. H., Pitman, N. C., Andino, J. E. G., Paredes, M. R., Heijink, B. M., & Torres‐Montenegro, L. A. (2021). A 5,000‐year vegetation and fire history for tierra firme forests in the Medio Putumayo‐Algodón watersheds, northeastern Peru. Proceedings of the National Academy of Sciences, 202022213. 10.1073/pnas.2022213118 PMC8501791 34580207

[ece38006-bib-0101] QGIS.org (2021). QGIS Geographic Information System. QGIS Association. http://www.qgis.org

[ece38006-bib-0058] Radhakrishna, S. (2018). Primate tales: Using literature to understand changes in human–primate relations. International Journal of Primatology, 39(5), 878–894. 10.1007/s10764-018-0035-9

[ece38006-bib-0059] Ramankutty, N., & Foley, J. A. (1999). Estimating historical changes in global land cover: Croplands from 1700 to 1992. Global Biogeochemical Cycles, 13, 997–1027. 10.1029/1999GB900046

[ece38006-bib-0060] Richard, A. F., Goldstein, S. J., & Dewar, R. E. (1989). Weed macaques: The evolutionary implications of macaque feeding ecology. International Journal of Primatology, 10(6), 569–594. 10.1007/BF02739365

[ece38006-bib-0061] Riley, E. P., & Priston, N. E. (2010). Macaques in farms and folklore: Exploring the human–nonhuman primate interface in Sulawesi, Indonesia. American Journal of Primatology, 72(10), 848–854. 20146249 10.1002/ajp.20798

[ece38006-bib-0062] Rivkin, L. R., Santangelo, J. S., Alberti, M., Aronson, M. F. J., de Keyzer, C. W., Diamond, S. E., Fortin, M.‐J., Frazee, L. J., Gorton, A. J., Hendry, A. P., Liu, Y., Losos, J. B., MacIvor, J. S., Martin, R. A., McDonnell, M. J., Miles, L. S., Munshi‐South, J., Ness, R. W., Newman, A. E. M., … Johnson, M. T. J. (2019). A roadmap for urban evolutionary ecology. Evolutionary Applications, 12(3), 384–398. 10.1111/eva.12734 30828362 PMC6383741

[ece38006-bib-0063] Rowe, N., & Myers, M. (2016). All the world's primates. The International Encyclopedia of Primatology; Encyclopedia published online at www.alltheworldsprimates.org

[ece38006-bib-0064] Seth, P. K., Seth, S., Chopra, P. K., & Reddy, G. J. (1989). Behavioural phylogeny of rhesus monkeys in India. In P. K. Seth, & S. Seth (Eds.), Perspectives on primate biology (pp. 219–243). Today’s & Tomorrow’s Printers.

[ece38006-bib-0065] Sigg, H., Stolba, A., Abegglen, J. J., & Dasser, V. (1982). Life history of hamadryas baboons: Physical development, infant mortality, reproductive parameters and family relationships. Primates, 23(4), 473–487. 10.1007/BF02373959

[ece38006-bib-0066] Sikes, R. S., & Animal Care and Use Committee of the American Society of Mammalogists (2016). 2016 Guidelines of the American Society of Mammalogists for the use of wild mammals in research and education. Journal of Mammalogy, 97(3), 663–688. 29692469 10.1093/jmammal/gyw078 PMC5909806

[ece38006-bib-0067] Sikes, R. S., Thompson, T. A., & Bryan, J. A. (2019). American Society of Mammalogists: Raising the standards for ethical and appropriate oversight of wildlife research. Journal of Mammalogy, 100(3), 763–773. 10.1093/jmammal/gyz019

[ece38006-bib-0068] Simons, E. A., Turley, K., & Frost, S. R. (2019). Phylogenetic perspectives on Catarrhine Talo‐Crural Joint Phenotypic Plasticity. The Anatomical Record, 302(11), 1977–1984. 10.1002/ar.24180 31120200

[ece38006-bib-0069] Smith, B. H., Crummett, T. L., & Brandt, K. L. (1994). Ages of eruption of primate teeth: A compendium for aging individuals and comparing life histories. American Journal of Physical Anthropology, 37(S19), 177–231. 10.1002/ajpa.1330370608

[ece38006-bib-0070] Sorrentino, R., Stephens, N. B., Carlson, K. J., Figus, C., Fiorenza, L., Frost, S., Harcourt‐Smith, W., Parr, W., Saers, J., Turley, K., Wroe, S., Belcastro, M. G., Ryan, T. M., & Benazzi, S. (2020). The influence of mobility strategy on the modern human talus. American Journal of Physical Anthropology, 171(3), 456–469. 10.1002/ajpa.23976 31825095

[ece38006-bib-0071] Stephens, L., Fuller, D., Boivin, N., Rick, T., Gauthier, N., Kay, A., Marwick, B., Armstrong, C. G., Barton, C. M., Denham, T., & Douglass, K. (2019). Archaeological assessment reveals Earth’s early transformation through land use. Science, 365(6456), 897–902. 31467217 10.1126/science.aax1192

[ece38006-bib-0072] Sterling, K. B. (2005). Early twentieth‐century mammal collecting in Africa: The Smithsonian‐Roosevelt East African Expedition of 1909–1910. Archives of Natural History, 32(1), 70–79. 10.3366/anh.2005.32.1.70

[ece38006-bib-0073] Tomiya, S., & Meachen, J. A. (2018). Postcranial diversity and recent ecomorphic impoverishment of North American gray wolves. Biology Letters, 14(1), 20170613. 10.1098/rsbl.2017.0613 29343558 PMC5803591

[ece38006-bib-0074] Turley, K., & Frost, S. R. (2018). Behaviorally induced change in post‐cranial (upper ankle joint) morphology: Phenotypic plasticity in an altered habitat. Human Evolution, 33(1), 31–46.

[ece38006-bib-0075] Turley, K., White, F. J., & Frost, S. R. (2015). Phenotypic plasticity: The impact of habitat and behavior (substrate use) on adult talo‐crural appositional articular joint shape both between and within closely related Hominoid species. Human Evolution, 30(1‐2), 49–67.

[ece38006-bib-0076] Walsh, P. D., Abernethy, K. A., Bermejo, M., Beyers, R., De Wachter, P., Akou, M. E., Huijbregts, B., Mambounga, D. I., Toham, A. K., Kilbourn, A. M., & Lahm, S. A. (2003). Catastrophic ape decline in western equatorial Africa. Nature, 422(6932), 611–614. 12679788 10.1038/nature01566

[ece38006-bib-0077] Zuccarelli, M. D. (2004). Comparative morphometric analysis of captive vs. wild African lion (Panthera leo) skulls. Bios, 75(4), 131–138.

